# Transforming and Tumorigenic Activity of JAK2 by Fusion to BCR: Molecular Mechanisms of Action of a Novel BCR-JAK2 Tyrosine-Kinase

**DOI:** 10.1371/journal.pone.0032451

**Published:** 2012-02-27

**Authors:** Álvaro Cuesta-Domínguez, Mara Ortega, Cristina Ormazábal, Matilde Santos-Roncero, Marta Galán-Díez, Juan Luis Steegmann, Ángela Figuera, Eva Arranz, José Luis Vizmanos, Juan A. Bueren, Paula Río, Elena Fernández-Ruiz

**Affiliations:** 1 Molecular Biology Unit, Hospital Universitario de la Princesa, Instituto de Investigación Sanitaria Princesa (IP) and Red Española de Investigación en Patología Infecciosa (REIPI), Madrid, Spain; 2 Department of Genetics, University of Navarra, Pamplona, Spain; 3 Department of Hematology, Hospital Universitario de la Princesa, Madrid, Spain; 4 Division of Hematopoiesis and Gene Therapy, Centro de Investigaciones Energéticas, Medioambientales y Tecnológicas (CIEMAT) and Centro de Investigación Biomédica en Red de Enfermedades Raras (CIBERER), Madrid, Spain; Emory University, United States of America

## Abstract

Chromosomal translocations in tumors frequently produce fusion genes coding for chimeric proteins with a key role in oncogenesis. Recent reports described a *BCR-JAK2* fusion gene in fatal chronic and acute myeloid leukemia, but the functional behavior of the chimeric protein remains uncharacterized. We used fluorescence *in situ* hybridization and reverse transcription polymerase chain reaction (RT-PCR) assays to describe a *BCR-JAK2* fusion gene from a patient with acute lymphoblastic leukemia. The patient has been in complete remission for six years following treatment and autologous transplantation, and minimal residual disease was monitored by real-time RT-PCR. *BCR-JAK2* codes for a protein containing the BCR oligomerization domain fused to the JAK2 tyrosine-kinase domain. *In vitro* analysis of transfected cells showed that BCR-JAK2 is located in the cytoplasm. Transduction of hematopoietic Ba/F3 cells with retroviral vectors carrying *BCR-JAK2* induced IL-3-independent cell growth, constitutive activation of the chimeric protein as well as STAT5 phosphorylation and translocation to the nuclei, where *Bcl-xL* gene expression was elicited. Primary mouse progenitor cells transduced with *BCR-JAK2* also showed increased proliferation and survival. Treatment with the JAK2 inhibitor TG101209 abrogated BCR-JAK2 and STAT5 phosphorylation, decreased *Bcl-xL* expression and triggered apoptosis of transformed Ba/F3 cells. Therefore, BCR-JAK2 is a novel tyrosine-kinase with transforming activity. It deregulates growth factor-dependent proliferation and cell survival, which can be abrogated by the TG101209 inhibitor. Moreover, transformed Ba/F3 cells developed tumors when injected subcutaneously into nude mice, thus proving the tumorigenic capacity of BCR-JAK2 *in vivo*. Together these findings suggest that adult and pediatric patients with *BCR-ABL*-negative leukemia and *JAK2* overexpression may benefit from targeted therapies.

## Introduction

Acute lymphoblastic leukemia (ALL) is a malignant disease characterized by the clonal expansion of lymphoid progenitors. Outcome is poorer in adults than in children, probably due to the higher frequency of Philadelphia (Ph') chromosome–positive ALL [1]. The Ph' chromosome results from a translocation, t(9;22)(q34;q11), which leads to the *BCR-ABL* fusion gene. One-third of adult ALL patients with the Ph' chromosome show major (M)-BCR rearrangements (resulting in a 210-kDa protein), whereas two-thirds have minor (m)-BCR rearrangements (resulting in a 190-kDa protein). These chimeric proteins are hyperactive tyrosine kinases (TK) that are located in the cytoplasm, where they recruit downstream effectors of cell proliferation and survival. Chromosomal rearrangements other than t(9;22)(q34;q11) are found in ALL, although they are less frequent (http://AtlasGeneticsOncology.org).

JAK2 is a non-receptor TK protein that is essential for signaling through a variety of cytokine receptors and is required for normal hematopoiesis [2], [3]. Activation of the JAK2-cytokine receptor complex leads to the recruitment and JAK2-mediated phosphorylation of STAT5 proteins whose subsequent dimerization and nuclear translocation induces target gene transcription [4]. The role of constitutively activated JAK2 or STAT5 in cellular transformation has been established [5]. Several fusion proteins involving the catalytic active JH1 domain of JAK2 have been reported to be associated with leukemia, as follows: 1) TEL-JAK2 was found in both pre-B-lineage and pre-T-lineage ALL and atypical chronic myelogenous leukemia (CML) [6], [7] 2) *PCM1-JAK2* gene fusion resulting from t(8;9)(p22;p24) in eosinophilia-associated atypical CML, ALL, acute myeloid leukemia (AML), and T cell lymphoma [8], and 3) *BCR-JAK2* gene fusion as the result of t(9;22)(p24;q11) was found in atypical CML [9], [10] and AML [11]. Other putative JAK2 translocations include *SSBP2-JAK2* in pre-B ALL [12], *PAX5*- and *STRN3-JAK2* in childhood ALL [13] and *SEC31A-JAK2* in classical non Hodgkin lymphoma [14]. Moreover, gain-of-function JAK2 mutations are common in myeloproliferative neoplasms [15] and in up to 15% of adult and high-risk pediatric B-ALL lacking *MLL*, *TCF3*, *TEL* and *BCR-ABL* rearrangements [16], [17], [18]. These observations have supported the search for selective inhibitors of JAK2 [19], [20] and several compounds are currently undergoing clinical trials for myelofibrosis [21], [22]. Here we provide for the first time evidence of the transforming and tumorigenic activity of JAK2 through fusion with BCR.

## Materials and Methods

### Ethics Statement

Patient written informed consent was obtained before bone marrow biopsies were taken. This protocol was carried out according to current Spanish legislation on clinical research in humans and was approved by the Hospital Universitario de la Princesa Clinical Investigation Ethics Committee (Approval ID: PI-424).

All experimentation with mice was carried out in accordance with institutional guidelines from the animal care and use committee from Centro de Investigaciones Tecnológicas y Medioambientales (CIEMAT) and approved by them with approval ID: HEM 4-09.

### Case description

A 58-year-old male presented with asthenia, abdominal pain and slight hepatosplenomegaly. The blood count was abnormal with anemia of Hb 10.9 g/dl and a platelet count of 41×10^3^/mm^3^. The white blood cell count was 10.8×10^3^/mm^3^ with 38% lymphocytes, 6% monocytes, 4% myelocytes and metamyelocytes, and 58% small lymphoblasts. Immunophenotyping of bone marrow disclosed B-lineage lymphoblasts (CD34^+^, CD19^+^, CD10^+^, MPO^−^, CD79a^+^, HLA-DR^+^, Tdt^+^, CD22^+^, and CD24^+^). Karyotyping performed on the marrow aspirate revealed 49,XY,+X,+2,+4,−9,−11,+19,add(19)(q13),+20,−22,+mar in 24 of 25 metaphases examined. *BCR-ABL* and *MLL* rearrangements were not detected by standard fluorescence *in situ* hybridization (FISH). While reverse transcriptase polymerase chain reaction (RT-PCR) analyses for *BCR-ABL* rearrangements were negative, we found a new *BCR-JAK2* transcript. The patient was diagnosed with ALL and started a standard high-risk ALL protocol. Hematologic, cytogenetic, and complete molecular remission (CMR) was achieved after five weeks, and the treatment proceeded through four standard consolidation cycles followed by an autologous peripheral blood stem cell transplant (SCT) with cyclophosphamide and total body irradiation (12 Gy). Low-dose interferon alpha was administered for five months as maintenance antileukemia therapy. More than six years later, the patient remains in complete remission.

### RT-PCR and real-time PCR

Total RNA was isolated using Ultraspec (Biotecx, Houston, TX, USA) and reverse transcribed using the Gene-Amp Gold RNA PCR Core Kit (Applied Biosystems, Cheshire, UK). PCR for BCR-ABL p190 was carried out according to [23]. BCR-B and JAK2-3 primers were used for *BCR-JAK2* qualitative PCR (Table 1). Long-range PCR to amplify full-length *BCR-JAK2* cDNA was performed using the GC-RICH PCR System (Roche Diagnostics, Basel, Switzerland) with BCR-Fw-T and JAK2-Rv-T primers. The PCR product was cloned in PCR2.1-TOPO (Invitrogen, Carlsbad, CA, USA) and sequenced using the d-rhodamine terminator cycle sequencing kit and an ABI PRISM 337 DNA sequencer (Applied-Biosystems). Sequences were aligned using BLAST and ClustalW applications (http://searchlauncher.bcm.tmc.edu/). *BCR-JAK2* was quantified using fluorescence resonance energy transfer (FRET)-hybridization probes designed by TibMolbiol (Berlin, Germany) and hybridized to a *BCR* sequence upstream of the *BCR-JAK2* breakpoint by means of BCR-B and JAK2-3 primers. The PCR master mix was prepared with 0.3 µM of each primer and probe using LightCycler FastStart DNA Master^PLUS^ HybProbe (Roche Diagnostics), and the reaction was performed in duplicate on a LightCycler 2.0 (Roche Diagnostics). Data were normalized using *BCR* levels in the same samples. Quantitative PCR (qPCR) for *Bcl-xL*, *Osm* was performed using the LightCycler FastStart DNA Master SYBR Green I kit (Roche Diagnostics) and *Socs2* (NM_007706) expression was analysed using RT^2^ qPCR primer assay (SABiosciences, Frederik, MD, USA) following the manufacturer's protocols. Data were normalized using *H3* (patient sample) or *Gapdh* (Ba/F3 cells) levels.

**Table 1:** Primers and probes used for PCR analysis.

PCR	Gene sequence	Name	5′- 3′	Annealing temperature (°C)	Cycles	Product size (bp)	Position (nt)
Qualitative	*BCR-JAK2*	BCR-Fw-T	GCCATGGTGGACCCGGTGG	55	35	2260	594–612^1^
		JAK2-Rv-T	AAGGTCATTTCTTTCATCCAGCC				3884–3906^2^
Quantitative	*BCR-JAK2*	FRET-probe-FL	GTCTTGCGGACGCCCACGA-FL *				1211–1229^1^
		FRET-probe-LC640	LC640-GGTGGCCTCGGACACGACAACC+				1188–1209^1^
		BCR-B-Fw	CCCCCGGAGTTTTGAGGATTG	63	45	997	1547–1567^1^
		JAK2-3-Rv	GGCCACAGAAAACTTGCTCTC				3576–3596^2^
	*BCR*	BCR-B-Fw	CCCCCGGAGTTTTGAGGATTG	63	45	301	1547–1567^1^
		BCR-B-Rv	ATCGTTGGGCCAGATCTGCC				1828–1847^1^
	*Bcl-xL*	Bcl-xL Fw	TCAGAGCTTTGAGCAGGTAGTG	58	45	187	726–747^3^ 552–573^4^
		Bcl-xL Rv	TCCCGTAGAGATCCACAAAAG				935–955^3^ 761–781^4^
	*Osm*	Osm Fw	AGAATCAGGCGAACCTCACGG	58	45	74	217–237 5
		Osm Rv	GTGTGTTCAGGTTTTGGAGGC				271–291^5^
	*Gapdh*	Gapdh Fw	AGAAGGTGGTGAAGCAGGCATC	58	45	116	820–841^6^
		Gapdh Rv	CGGCATCGAAGGTGGAAGAGTG				915–936^6^
	*H3*	H3 Fw	AAAGCCGCTCGCAAGAGTGCG	62	35	201	185–205^7^
		H3 Rv	ACTTGCCTCCTGCAAAGCAC				387–406^7^

Fw, forward; Rv, reverse; FL, fluorescein; LC640, LightCycler Red.

*,+: inverse and complementary probe sequences to human *BCR*;

1 human *BCR* (NM_004327);

2 human *JAK2* (NM_ 004972);

3 human *Bcl-xL* (*BCLXL* or *BCL2L1*) (NM_ 1385781);

4 mouse *Bcl-xL* (*BCL2-like 1* or *Bcl2l1*) (NM_ 009743) and

5 mouse *Osm* (oncostatin M) (NM_001013365.2) [39];

6 mouse *Gapdh* (glyceraldehyde-3-phosphate dehydrogenase) NM_008084.2),

7 human H3 (*H3F3A*) (NM_002107.3).

Primers for *Bcl-xL* and *H3F3A* are designed to hybridize with both human and mouse sequences (patient samples and Ba/F3 cells, respectively).

### Fluorescence *in situ* hybridization

**Figure 1:**
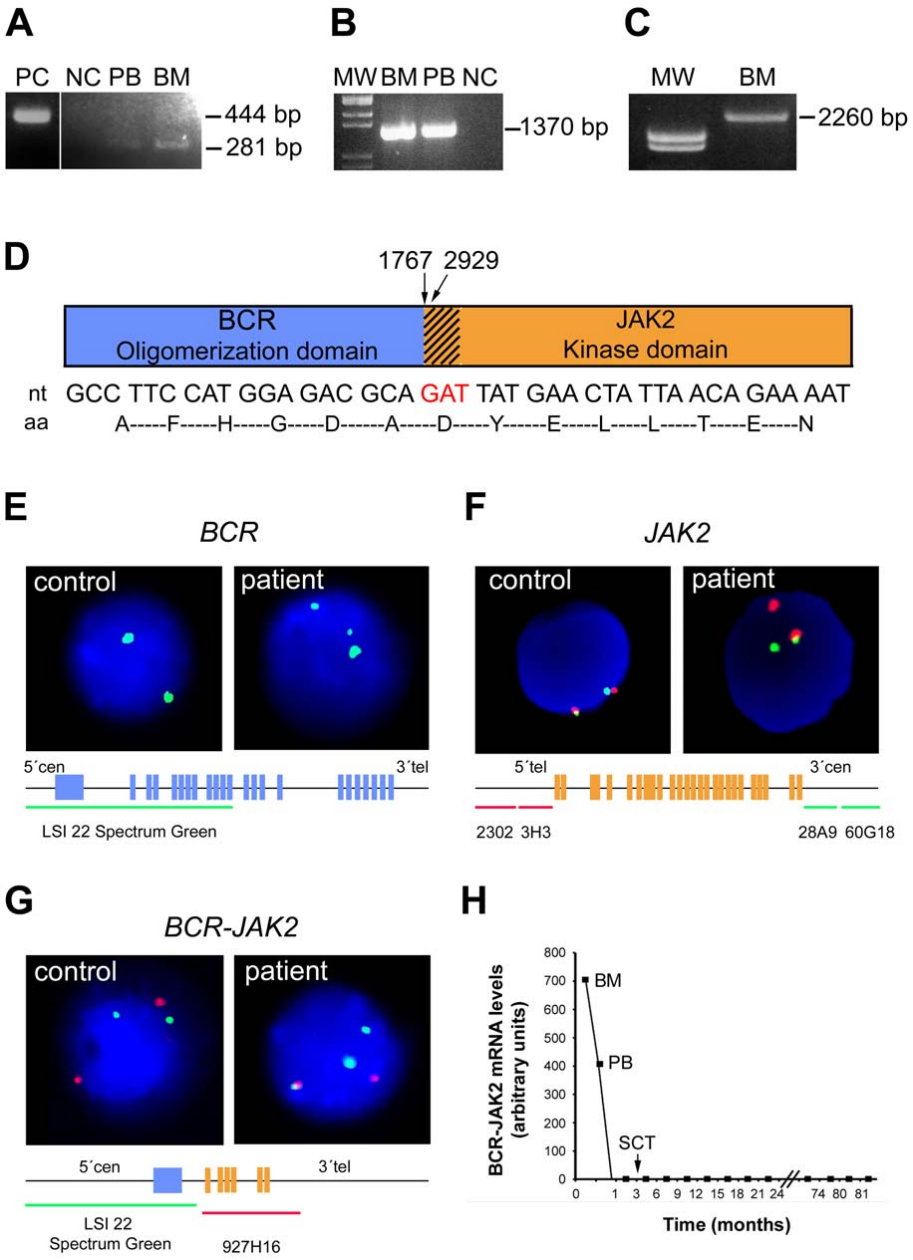
Cloning and identification of BCR-JAK2 fusion gene. (**a**) Nested RT-PCR analysis with standard primers for p190 *BCR-ABL* amplification (BCR-B and ABL3) on the ALL patient's samples at diagnosis (PB, peripheral blood; BM, bone marrow) showing an unexpected PCR product of 281 bp. PC: positive control (BCR-ABL^+^ p190); NC, healthy negative control. (**b**) Verification of the *BCR-JAK2* fusion gene by RT-PCR analysis using primers for *BCR* (BCR-B) and for the region flanking the stop codon of *JAK2* (JAK2-Rv-T). NC, BCR-ABL^+^ p210; MW, molecular weight marker. The 1,370-bp product contains part of *BCR* exon 1 fused to exon 19 of *JAK2* tyrosine-kinase. (**c**) Full-length *BCR-JAK2* cDNA amplification. RT-PCR with primers extending the start codon of *BCR* and the stop codon of *JAK2*. (**d**) Schematic diagram of the putative BCR-JAK2 fusion protein (strips represent the fragment of the JAK2 domain JH2). Nucleotide (nt) and amino acid (aa) sequences below showed the *BCR-JAK2* breakpoint region (red lettering represents the common *BCR* and *JAK2* triplet). Numbers indicate the nucleotide breakpoint position: 1767 for the *BCR* gene (NM_004327) and 2929 for *JAK2* (NM_004972). (**e**) Interphase nuclei of bone marrow cells at diagnosis after FISH analysis with a probe extending the 5′ region of *BCR*, showing three green signals indicating a translocation affecting one *BCR* allele. Schematic *BCR* gene structure and probe localization are represented below the nuclei. (**f**) FISH analysis with BAC probes flanking *JAK2*. Co-localized green and red signals correspond to the normal *JAK2* copy. Rearranged *JAK2* allele is detected in the patient's nucleus as single green and red signals. (**g**) FISH analysis for *BCR-JAK2* translocation. Two *BCR* (green) and two *JAK2* (red) signals were observed in the control, whereas three BCR signals were detected in the patient, one of them fused with one red signal, revealing fusion between *BCR* and *JAK2*. (**h**) *BCR-JAK2* qPCR. Specific FRET-hybridization probes were used. Arbitrary values for *BCR-JAK2* transcripts from diagnosis up to the present time are represented. Data were normalized with *BCR*. Molecular response was achieved before SCT. BM, bone marrow; PB, peripheral blood.

FISH was performed on bone marrow interphase nuclei using a probe set for *BCR* (LSI® 22 BCR Spectrum Green™; Vysis, Downers Grove, IL, USA) and bacterial artificial chromosome (BAC) clone flanking probes covering the whole *JAK2* gene (see probe's names and locations in Figure 1, E–G). *JAK2* clone positions were based on data provided by the University of California–Santa Cruz Genome Browser (http://genome.ucsc.edu) version hg18 (March 2006). BAC clones were obtained from the BACPAC Resource Center at the Children's Hospital (Oakland, CA, USA) and from the Sanger Institute Mapping Core Group at The Wellcome Trust Sanger Institute (Hinxton, United Kingdom) (http://cancerres.aacrjournals.org). BAC DNA was isolated from 5-ml cultures using a standard miniprep procedure (PhasePrep™ BAC DNA Kit, Sigma-Aldrich, St. Louis, MO, USA) and labelled by nick translation with SpectrumGreen-dUTP and SpectrumOrange-dUTP (Vysis). FISH was performed according to standard procedures. In all experiments, 100–150 interphase nuclei were scored, and no metaphases were observed.

### Cell lines and reagents

HEK293T cells (ATCC) were cultured in DMEM (Cambrex, East Rutherford, NJ, USA) containing 10% fetal calf serum, 100 U/ml penicillin, 100 µg/ml streptomycin, 50 µg/ml gentamicin and 4 mM L-glutamine at 37°C and 5% CO_2_. Murine Ba/F3 pro-B cells (ATCC) were grown in DMEM with 10 ng/ml of recombinant IL-3 (R&D, Minneapolis, MN, USA). Cytokine-independent Ba/F3 cells transduced with *TEL-JAK2* were a gift from Dr. G. Reuther (Florida University, Tampa, USA). JAK2 inhibitor TG101209 was provided by Dr. Fuller (TargeGen Inc., San Diego, CA, USA). Stock solutions were prepared in dimethylsulfoxide (DMSO).

### DNA constructs and retroviral vectors

For expression in HEK293T cells, *BCR-JAK2* cDNA was subcloned into the expression vector pcDNA3.1 (Invitrogen) through an *Eco*R I site. For retroviral infections in Ba/F3 cells, cDNA was cloned into the *Eco*R I site of the pLZR CMV-IRES-EGFP (pLZR) vector (Dr. Bernad, Centro Nacional de Investigaciones Cardiovasculares, Madrid, Spain) to obtain the pLZR-BJ-IRES-EGFP (pLZR-BJ) vector. The pLZR vector was used as a control. All constructs were verified by DNA sequencing.

### Cell transfections, retrovirus production, infection, and proliferative assays

Transient transfections in HEK293T cells were performed with 3 µg of plasmid in Opti-MEM (Lonza, Basel, Switzerland) using Lipofectin (Invitrogen) according to the manufacturer's instructions and analyzed 48 h later. For Ba/F3 cell infection, retroviral supernatants were obtained as previously described [24] after transient transfection of Nxe cells with 10 µg of the pLZR vector bearing or not *BCR-JAK2*. At 24 and 48 h post-transfection, viral supernatants were harvested, filtered, and stored at −80°C. Ba/F3 cells were infected with retroviral supernatants preloaded four times during 30 min in Retronectin (12.5 mg/ml, Takara Bio Inc., Shiga, Japan)-coated plates. After 7 days, EGFP+ cells were sorted by flow cytometry using a FACSAria cytometer (Becton-Dickinson, Franklin Lanes, NJ, USA). For proliferative assays, 3×10^5^ cells of Ba/F3-mock or Ba/F3-BJ cells were cultured with or without IL-3 (10 ng/ml) on 24-well plates and counted daily by trypan blue exclusion. TG101209 dose-dependent kinetic assays were performed as described [19]. Lineage negative (Lin-) progenitor cells were selected from male Balb/c mice. Total bone marrow cells obtained by perfusion of tibiae and femur with IMDM medium (Invitrogen) were counted and negatively selected by MACS Lineage Cell Depletion Kit (Miltenyi Biotec, Bergisch Gladbach, Germany) following manufacturer instructions. Lin- cells were prestimulated during 48 h (100 ng/µl mSCF, 100 ng/µl hIL-11) as previously described [25] and seeded in retronectin-coated 6-well plates for retroviral transduction either with pLZR (mock) or pLZR-BJ particles. Two cycles of retroviral transduction with a multiplicity of infection of 50 virus/cell were conducted, and cells were finally washed and seeded in 6-well plates at 5×10^4^ cells/ml for in vitro expansion. Cells were counted by trypan blue exclusion every week during 30 days.

### Immunofluorescence

See Methods S1.

### Western blot analysis

Cells were treated with lysis buffer (0.1 M Tris pH 8, 0.3 M NaCl, 2% NP-40, 1X EDTA-free Protease Inhibitor Cocktail and 1X PhosSTOP Phosphatase Inhibitor Cocktail [Roche]). Whole cell lysates (50 µg) or anti-phosphotyrosine immunoprecipitates (pTyr, BD Biosciences) (500 µg) were resolved by SDS-PAGE under reducing conditions and blotted with the following antibodies (Ab's): anti-JAK2, anti-p-STAT5 (Cell Signaling, Beverly, CA, USA), anti-STAT5, anti- IκBα (Santa Cruz Biotechnology, Santa Cruz, CA, USA), anti-Bcl-xL (BD Biosciences), anti-tubulin (Sigma-Aldrich), anti-TBP (Abcam, Cambridge, UK) and secondary HRP-conjugated anti-mouse/anti-rabbit antibodies (GE Healthcare, Bucks, UK). Enriched nuclear and cytoplasmic extracts were obtained using an NE-PER extraction reagents kit (Thermo Scientific, Rockford, IL) after 10 min of cold hypotonic shock in 10 mM NaCl. Western blots were visualized using SuperSignal West Pico Chemiluminescent Reagent (Thermo Scientific). Densitometry was performed using Image-Gauge v3.46 software (FujiFilm, Tokyo, Japan).

### Luciferase reporter assay

See Methods S1.

### Apoptosis analysis by flow cytometry

Apoptosis was measured by the binding of Annexin-V and the incorporation of 7-amino-actinomycin D (7-AAD) after treatment with the inhibitors. Cells were washed twice with cold PBS and then resuspended in 1X Binding Buffer (BD Biosciences) at a concentration of 1×10^6^ cells/ml. Staining with annexin V-phycoerythrin (PE) and 7-AAD was performed following the manufacturer's instructions (BD Biosciences). Samples were analyzed through flow cytometry using an EPICS XL cytometer (Beckman Coulter, Fullerman, CA, USA).

### Tumorigenicity assays in nude mice

Ten female BALB/c OlaHsd-Foxn1^nu/nu^ mice (Harlan Laboratories Inc., Indianapolis, IN, USA) were subcutaneously injected with 10^7^ Ba/F3 cells expressing BCR-JAK2 together with EGFP (right flank) and 10^7^ Ba/F3-mock cells expressing only EGFP (left flank). Mice were examined by palpation for tumor formation for up to 20 days and then sacrificed by cervical dislocation and analyzed in a 2000-MM Image Station (Kodak, Rochester, NY, USA) to detect an EGFP signal. Tumors were extracted and disaggregated with 20 µg/ml of collagenase IMDM (Sigma-Aldrich) for 4 h prior to RNA extraction or flow cytometry analysis in an EPICS XL (Beckman Coulter).

## Results

### *BCR-JAK2* fusion gene identification and real–time PCR analysis for minimal residual disease follow–up

Standard RT-PCR was performed to detect *BCR-ABL* using RNA from bone marrow and peripheral blood samples at diagnosis. The result for the M-*BCR-ABL* breakpoint region was negative (data not shown), but for m-*BCR-ABL* the reaction revealed a smaller product compared with the positive control (Figure 1A). Sequence analysis revealed that this in-frame product corresponded to the expected *BCR* exon 1 region, which is usually found in the p190 *BCR-ABL* transcript fused to 90 bp corresponding to *JAK2* exon 19 and flanked by the *ABL* reverse primer (ABL3) (Figure S1). In line with the results of recent cases of CML [9], [10] and AML [11], these data suggested the presence of a *BCR-JAK2* fusion gene. RT-PCR assays were performed on the same samples (Figure 1B) with primers for BCR (BCR-B) and the stop codon of JAK2 (JAK2-Rv-T, see Table 1). A specific band of 1,307 bp was obtained and sequenced, containing BCR and the TK domain of JAK2. The full-length *BCR-JAK2* cDNA was amplified using primers flanking the start codon of *BCR* (BCR-Fw-T) and the stop codon of *JAK2* (JAK2-Rv-T). A specific band of 2,260-bp was obtained (Figure 1C), sequenced and subcloned. The complete cDNA encoded a putative 749 amino acid polypeptide with a predicted molecular mass of 83 kDa that contained the N-terminal coiled-coil domain of *BCR* followed by the TK domain of *JAK2* (JH1) (Figure 1D).

An additional FISH analysis was performed (Figure 1E–G). Using a 300-kb green probe extending the 5′ *BCR* gene region, 3 signals were detected in 73 of 100 nuclei scored, suggesting that one *BCR* allele is broken (Figure 1E). In addition, when specific 5′ red and 3′ green *JAK2* flanking probes were used, three signals were observed in 62 of 101 nuclei scored: one co-localized green-red signal corresponding to the normal gene copy, and one red signal and one green signal, suggesting a breakpoint within the *JAK2* locus (Figure 1F). Finally, hybridization with a green probe for *BCR* and a red probe for *JAK2* showed three green and two red signals. One of the green split signals was fused with one red signal, revealing a fusion between the *BCR* and *JAK2* genes in 62 out of 150 (41.3%) nuclei scored (Figure 1G). A specific *BCR-JAK2* real-time PCR assay was designed for follow-up of minimal residual disease (MRD). The patient acquired CMR five weeks after induction therapy and prior to SCT, and CMR was maintained to the present time (more than six years) (Figure 1H).

### Ectopic expression of BCR-JAK2 renders hematopoietic cells growth factor–independent

**Figure 2:**
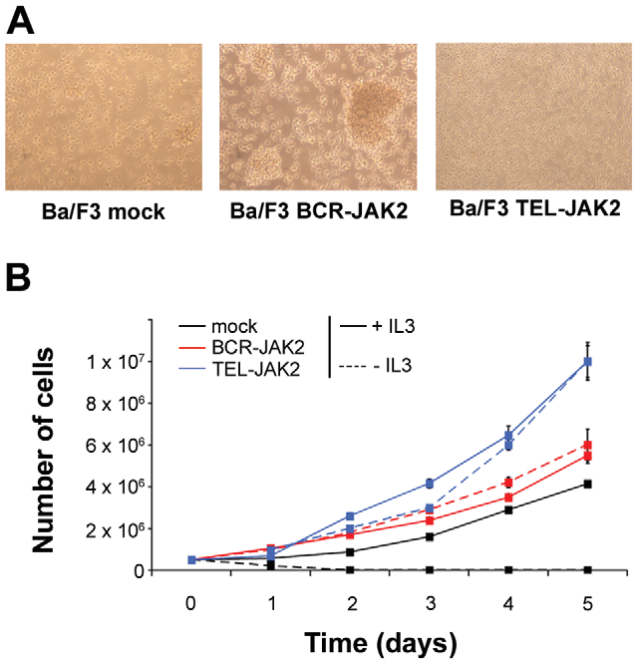
BCR-JAK2 renders Ba/F3 cells growth factor-independent. Ba/F3 cells were transduced in three independent experiments with pLZR-IRES-EGFP (pLZR) retroviral vector alone (Ba/F3-mock) or pLZR carrying BCR-JAK2 (Ba/F3-BCR-JAK2). Ba/F3 cells transduced with TEL-JAK2 were used as positive control (Ba/F3-TEL-JAK2). (**a**) Microphotographs of cultured Ba/F3-mock growing with IL-3 and Ba/F3-BCR-JAK2 and Ba/F3-TEL-JAK2 cells without IL-3 (×20 magnification). (**b**) For proliferation assays, 3×10^5^ cells were incubated in the presence or absence of 10 ng/ml IL-3 for 5 days. Viable cells were counted daily by trypan blue exclusion. Ba/F3-BCR-JAK2 cells proliferate either in the presence or absence of IL-3. Results are given as mean ± SD (n = 3).

To investigate the transforming potential of BCR-JAK2, the interleukin-3 (IL-3)-dependent Ba/F3 cell line was used as a model [26] and transduced with the pLZR-IRES-EGFP retroviral vector containing *BCR-JAK2* cDNA (Ba/F3-BCR-JAK2) or the control vector (Ba/F3-mock). As a consequence of BCR-JAK2 expression, IL-3-independent Ba/F3 cells were obtained after 2–4 days of culture, yet no independent cells were obtained with the control vector. Interestingly, Ba/F3-BCR-JAK2 cells showed a cluster-forming growth pattern when compared to Ba/F3-mock cells (Figure 2A). Proliferative assays were developed with Ba/F3-BCR-JAK2 and Ba/F3-mock cells growing either in the presence or absence of IL-3 (Figure 2B). Ba/F3-BCR-JAK2 cells proliferated at a comparable rate in both conditions and this rate was even higher than Ba/F3-mock cells growing with IL-3. Cytokine-independent Ba/F3 cells transduced with *TEL-JAK2* were used as a positive control because of its in vitro transforming ability [27], [28]. These data suggest that BCR-JAK2 conferred a proliferative advantage and transformed hematopoietic cells in a similar way to TEL-JAK2 and other BCR-TK chimeric proteins such as BCR-ABL, BCR-PDGFRA, or BCR-FGFR1 [29], [30], [31].

### BCR-JAK2 is constitutively phosphorylated, triggering STAT5 activation which translocates to the nucleus and induces *target gene* expression on transduced Ba/F3 cells

**Figure 3:**
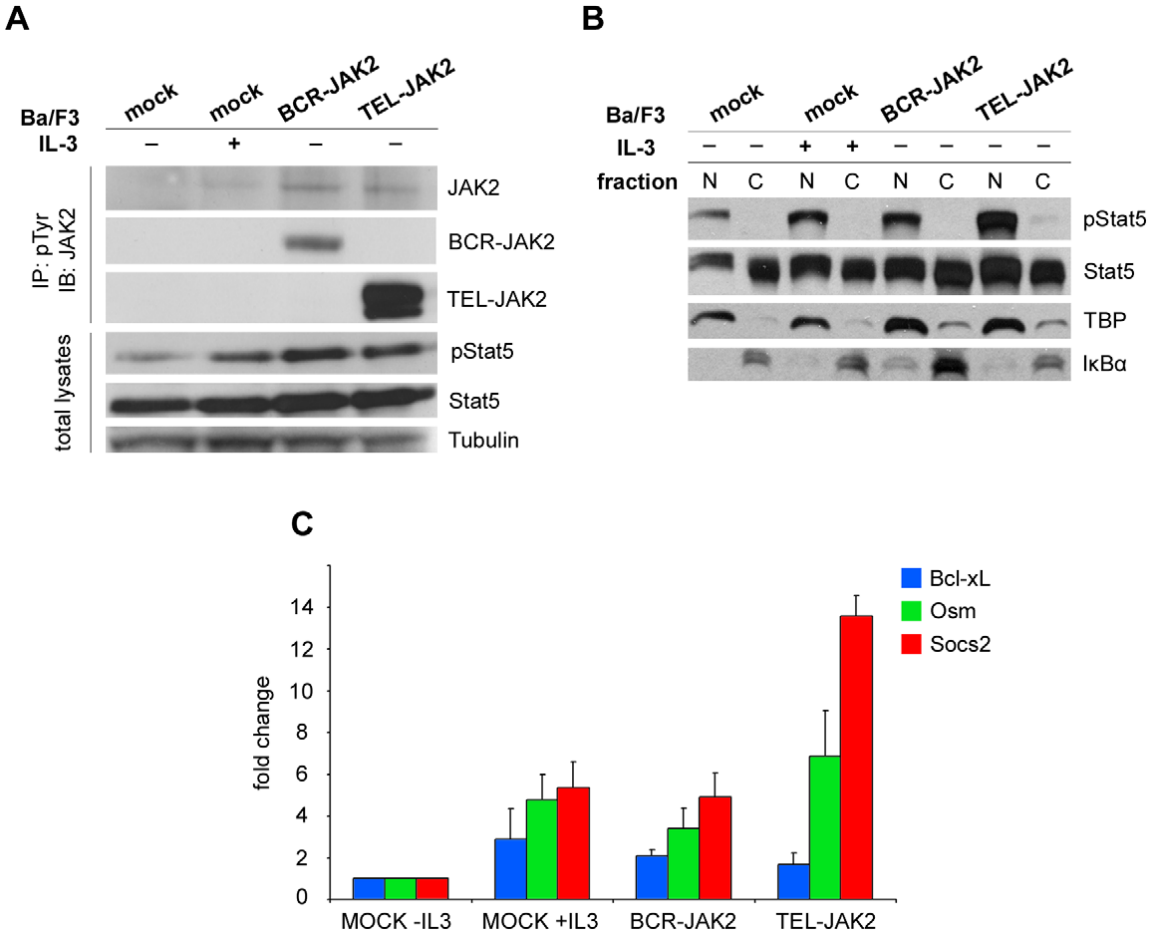
BCR-JAK2 is constitutively phosphorylated, triggering STAT5 activation which translocates to the nucleus and induces STAT5 target gene expression on transduced Ba/F3 cells. (**a**) Western blot analysis of Ba/F3 cells infected with BCR-JAK2, TEL-JAK2 or vector alone (mock). Cellular lysates were immunoprecipitated with anti-pTyr Ab and immunoblotted with anti-JAK2 Ab to see pJAK2 (upper panel: endogenous JAK2: 130 kDa, BCR-JAK2: 90 kDa, TEL-JAK2: 72 kDa). TEL-JAK2 protein expression is higher than BCR-JAK2 in transduced Ba/F3 cells. Whole cell lysates were probed with anti-pSTAT5 and anti-STAT5 Ab's (bottom). The expression levels of Tubulin were used as a loading control. (**b**) Enriched cytoplasmic (C) and nuclear (N) extracts from Ba/F3-mock,Ba/F3-BCR-JAK2 and Ba/F3-TEL-JAK2 cells were prepared from total cell lysates and blotted with anti-pSTAT5 Ab. TATA-Binding protein (TBP) and IkBα were used as loading controls for enriched nuclear and cytoplasmic fractions, respectively. (**c**) Expression of *Bcl-xL, Osm and Socs2* in Ba/F3-mock, Ba/F3-BCR-JAK2 and Ba/F3-TEL-JAK2 cells by qPCR. For comparative purposes, mRNA levels in untreated cells were normalized to 1. Bars represent fold changes of each gene normalized using *GADPH* levels. Results are given as mean ± SEM (n = 3).

To test whether Ba/F3 cells stably transduced with pLZR-BJ expressed tyrosine-phosphorylated BCR-JAK2 and activated STAT5, cellular lysates were immunoprecipitated with anti-pTyr Ab and immunoblotted with anti-JAK2 to detect pJAK2. A tyrosine-phosphorylated product of approximately 90 kDa corresponding to the chimeric protein was detected in Ba/F3-BCR-JAK2 cells, whereas no such product was seen in Ba/F3-mock cells (Figure 3A). Therefore, BCR-JAK2, as TEL-JAK2 [6], was expressed and constitutively phosphorylated on transduced cells. It is worth noting that TEL-JAK2 protein expression is higher than BCR-JAK2 in transduced-Ba/F3 cells and this could explain their higher growth rate depicted in figure 2B. To investigate whether BCR-JAK2 induced STAT5 activation, Western blot was performed using anti-pSTAT5 and anti-STAT5 Ab's, revealing STAT5 phosphorylation in all Ba/F3 cells tested, with increased p-STAT5 levels in Ba/F3-mock cells growing with IL-3 and Ba/F3-BCR-JAK2 and -TEL-JAK2 transduced cells (Figure 3A). When enriched cytoplasmic and nuclear fractions from total cell lysates were isolated, pSTAT5 was detected in the enriched nuclear fraction of these three cell types, suggesting functional STAT5 activation (Figure 3B). To investigate the subcellular localization of BCR-JAK2, transiently transfected HEK293T cells were analyzed by immunofluorescence with anti-JAK2 Ab. No signal was detected on HEK293T-mock cells, whereas HEK293T-BCR-JAK2 transfected cells showed intense staining for JAK2 in the cytoplasm (Figure S2). Taken together, these results suggest that BCR-JAK2 is located in the cytoplasm and induces cytokine-independent STAT5 phosphorylation and translocation into the nucleus.

To demonstrate STAT5 biological activity, qPCR of *Bcl-xL* gene expression was performed on transduced Ba/F3 cells (Figure 3C), given its upregulation upon STAT5 activation in response to cytokines [32], [33], [34] and leukemic fusion proteins [35], [36]. Two to three-fold upregulation in *Bcl-xL* expression was detected in Ba/F3-BCR-JAK2 and Ba/F3-mock cells growing with IL-3 suggesting cytokine-independent BCR-JAK2-mediated upregulation of *Bcl-xL*. Moreover, the quantification of *Bcl-xL* using RNA from the patient sample at diagnosis revealed increased expression of this gene compared with samples in CMR (Figure S3). In addition, luciferase reporter assays in HEK293T-BJ cells co-transfected with a plasmid bearing the promoter region of *Bcl-xL* containing a STAT binding element showed that BCR-JAK2 induced *Bcl-xL* promoter activation (Figure S4). As expected, HEK293T-mock cells were unable to induce *Bcl-xL* activation, whereas HEK293T cells transfected with a constitutively active STAT5 construct showed enhanced *Bcl-xL* activation. Therefore, we conclude that BCR-JAK2 elicited *Bcl-xL* promoter activation.

In addition to *Bcl-xL* expression, we have investigated other STAT5 target genes as oncostatin M (*Osm*) and suppressor of cytokine signaling-2 (*Socs2*) [27], [37], [38], [39] (Figure 3C). As expected, both genes had increased expression in Ba/F3-BCR-JAK2, TEL-JAK2 [27] and mock cells growing with IL-3.

### TG101209 abrogated BCR-JAK2 activation and STAT5 phosphorylation leading to downregulation of STAT5 target gene transcription

**Figure 4:**
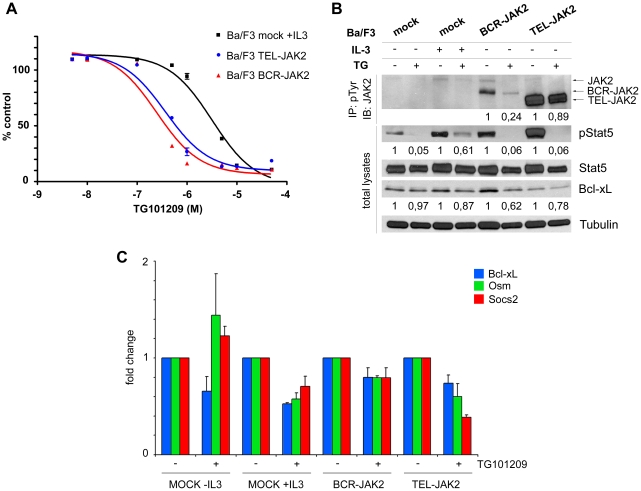
TG101209, a JAK2 inhibitor, down-regulates JAK2, BCR-JAK2, and STAT5 tyrosine-phosphorylation, as well as target gene expression of Ba/F3-BCR-JAK2 transduced cells. (**a**) Sigmoidal dose-response curve showing viability of Ba/F3-mock growing with IL-3 (IC_50_ = 3180 nM) and Ba/F3-BCR-JAK2 and Ba/F3-TEL-JAK2 growing in absence of IL-3 (IC_50_ = 246 and 369 nM, respectively). The percentage of growth, relative to that of cells in the absence of drug, is plotted for increasing concentrations of TG101209 (where X axis is the logarithm of concentration). (**b**) Western blot analysis of transduced Ba/F3 cells treated for 12 h with 1 µM TG101209 (TG) or the vehicle (DMSO). Cellular lysates were immunoprecipitated with anti-pTyr Ab and immunoblotted with anti-JAK2 (upper panel). Whole cell lysates were probed with anti-pSTAT5, anti-STAT5 and anti-Bcl-xL Ab's (bottom). The expression levels of Tubulin were used as a loading control. STAT5 levels were used as a loading control for pSTAT5. (**c**) qPCR for *Bcl-xL, Osm and Socs2* expression on Ba/F3-mock, Ba/F3-BCR-JAK2 and Ba/F3-TEL-JAK2 cells treated for 12 h with 1 µM TG101209. For comparative purposes, mRNA levels in untreated cells were normalized to 1. Bars represent fold changes of each gene after normalization with *GADPH* levels. Samples from three independent experiments were measured. Results are given as mean ± SEM (n = 3).

TG101209 is a potent and selective inhibitor of JAK2 (IC_50_ = 6 nM) that induces cell cycle arrest and apoptosis in Ba/F3 cells expressing the JAK2V617F mutation (IC_50_ = 170 nM) and in JAK2V617F-expressing acute myeloid leukemia cells through the inhibition of pJAK2V617F and pSTAT5 [19]. Therefore, Ba/F3 cells expressing BCR-JAK2, TEL-JAK2 or Ba/F3-mock cells were treated with TG101209 for 12 h and compared with the vehicle DMSO-treated cells. To analyze cell viability, dose-dependent kinetic assays were performed and 1 µM TG101209 dose was selected (Fig. 4A). Interestingly, this assay revealed a higher susceptibility of Ba/F3-BCR-JAK2 and Ba/F3-TEL-JAK2 cells to TG101209 compared with Ba/F3-JAK2V617F cells [19]. Cellular lysates were immunoprecipitated with anti-pTyr Ab and immunoblotted with anti-JAK2 to detect pJAK2 (Figure 4B, upper panel). Densitometry analysis revealed a 76% inhibition rate of BCR-JAK2 phosphorylation at 12 h of treatment compared to 10% inhibition of pTEL-JAK2 but this could be due to the higher TEL-JAK2 expression. Endogenous JAK2 phosphorylation was also inhibited, as previously described [19]. However, phosphorylation of endogenous JAK2 is not seen or inconsistently detected in Ba/F3-BCR-JAK2 cells as it has been on Ba/F3 cells transduced with TEL-JAK2 [27], [28], [40], [41]. Whole cell lysates from the same experiment were used and probed with anti-pSTAT5 Ab to detect whether BCR-JAK2 inhibition impaired STAT5 activation (Figure 4A, lower panel). STAT5 phosphorylation on Ba/F3-BCR-JAK2, Ba/F3-TEL-JAK2 and IL-3-starved Ba/F3-mock cells was almost completely inhibited, whereas Ba/F3-mock cells growing with IL-3 showed 40% inhibition. Bcl-xL expression was inhibited by 40% on TG101209-treated Ba/F3-BCR-JAK2 cells whereas on Ba/F3-mock and Ba/F3-TEL-JAK2 cells this inhibition was lower. qPCR for *Bcl-xL, Osm and Socs2* transcript detection was performed on Ba/F3-mock, Ba/F3-BCR-JAK2 and Ba/F3-TEL-JAK2 cells treated with TG101209 for 12 h. (Figure 4C). Both treated BCR-JAK2 and TEL-JAK2-expressing cells showed inhibition of *Bcl-xL, Osm and Socs2* expression. Taken together, these data suggest that TG101209 inhibited STAT5 activation and STAT5-mediated induction of *Bcl-xL, Osm and Socs2* through inhibition of both chimeric proteins in transduced Ba/F3 cells and inhibition of endogenous JAK2 in Ba/F3-mock cells growing with IL-3.

### TG101209 induces Ba/F3-BCR-JAK2 cell death by apoptosis

**Figure 5:**
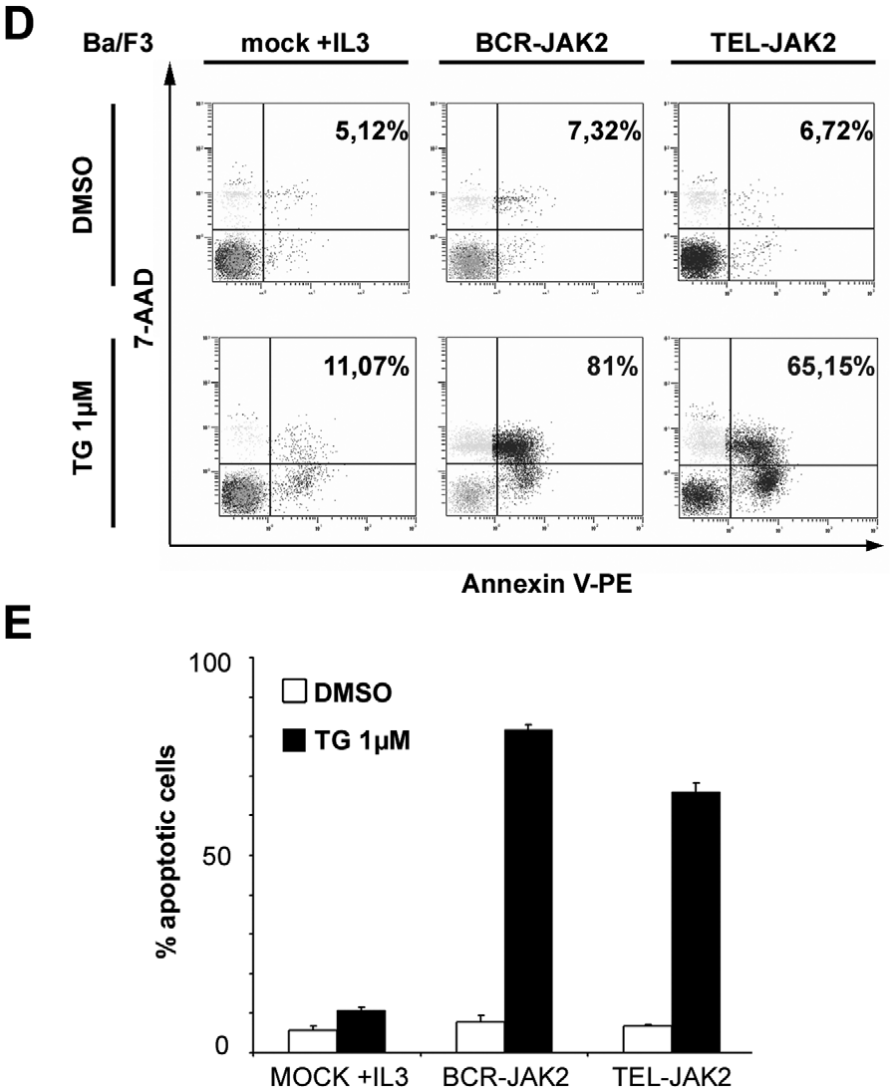
TG101209 induces apoptosis of Ba/F3-BCR-JAK2 cells. Ba/F3-mock growing with IL-3, Ba/F3-BCR-JAK2 and Ba/F3-TEL-JAK2 cells (10^5^/ml) were grown in 24-well plates and treated either with DMSO or 1 µM TG101209 (TG) for 24 h. Ba/F3-mock cells growing for 24 h without IL-3 were dead (data not shown). Apoptosis was measured using flow cytometry analysis as the percentage of cells expressing annexin-V/7-AAD on the cell surface. (a) One representative experiment out of three is shown. (b) Quantification of apoptotic cells. Results are given as mean ± SEM (n = 3).

To determine whether TG101209 inhibitor induced apoptosis of BCR-JAK2 and TEL-JAK2-expressing cells, as previously described for cell lines bearing the JAK2V167F mutation [19], Annexin-V binding assays were performed using flow cytometry (Figure 5A). Treatment of Ba/F3-BCR-JAK2 cells with TG101209 (1 µM) for 24 h resulted in an increase of approximately 80% in Annexin-V/7-AAD–positive cells. As expected, TG101209 only showed a modest increase in apoptosis of Ba/F3 mock cells growing with IL-3 (Figure 5B), thus demonstrating that induction of apoptosis in Ba/F3-BCR-JAK2 cells was a consequence of BCR-JAK2 inhibition. The rate of apoptotic Ba/F3-TEL-JAK2-treated cells was slightly lower than Ba/F3-BCR-JAK2-treated cells in accordance with their reduced susceptibility to TG101209 (Figure 4A). This could be due to higher levels of TEL-JAK2 protein expression (Figures 3A and 4B).

### Nude mice injected with Ba/F3 cells expressing BCR-JAK2 develop tumors

**Figure 6:**
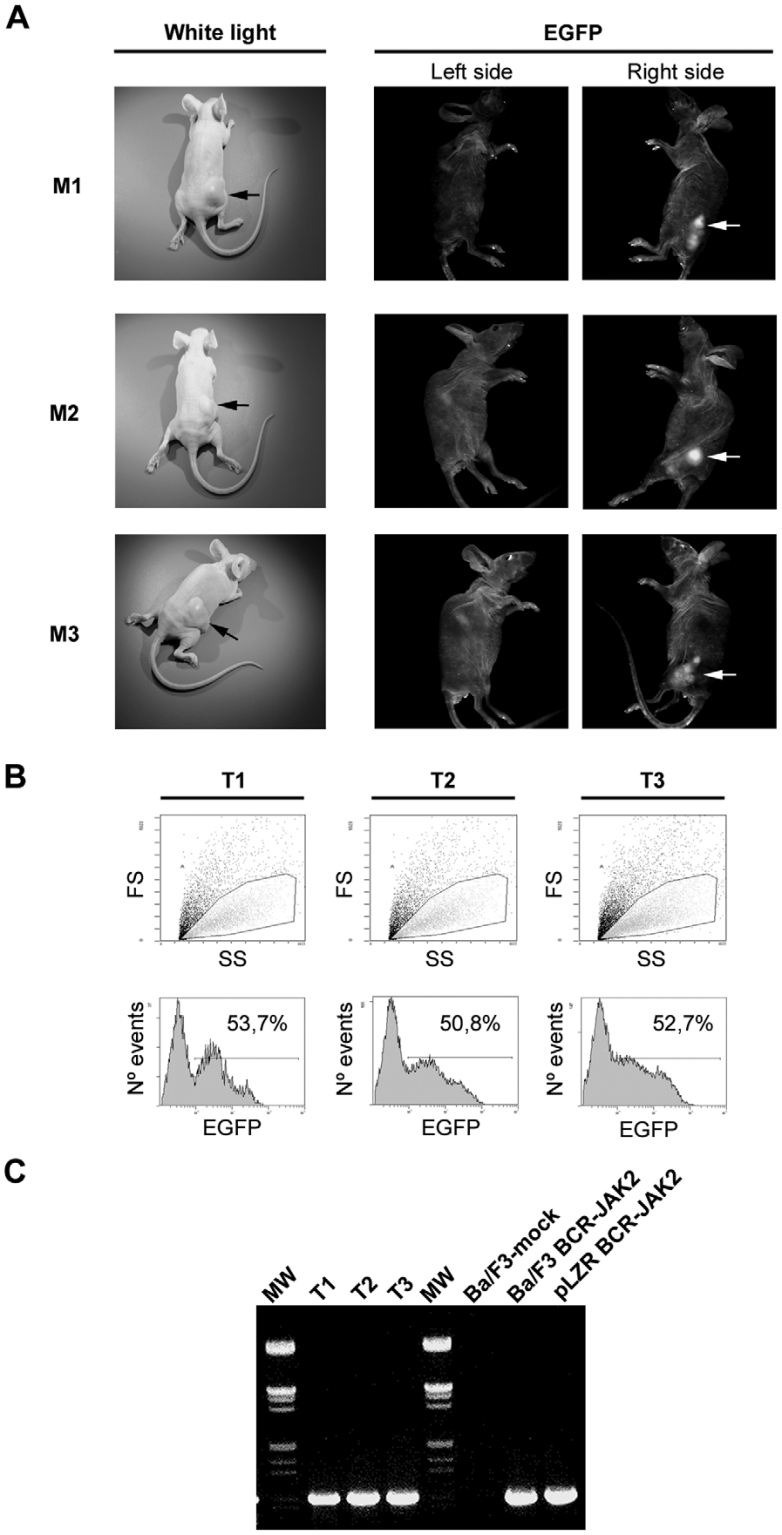
Nude mice injected with Ba/F3-BCR-JAK2 cells develop tumors. Mice were subcutaneously injected with 10^7^ Ba/F3-mock cells (left flank) and Ba/F3-BCR-JAK2 cells (right flank). (**a**) Left panel: white light photograph showing mice bearing a tumor only on the right side, where Ba/F3-BCR-JAK2 cells were injected (black arrows). Right panel: EGFP^+^ tumor (white arrows) captured on a digital photostation showing the reflected image of the mice (right side). No EGFP expression was detected when Ba/F3-mock cells were injected (left side). (**b**) Flow cytometry analysis of EGFP-expressing tumor cells. (**c**) RT-PCR analysis to detect BCR-JAK2 expression using BCR-B and JAK2-3 primers. RNA from three different tumors was analyzed compared with RNA from Ba/F3-mock and Ba/F3-BCR-JAK2 cells used as negative and positive controls, respectively. pLZR-BCR-JAK2: plasmid bearing *BCR-JAK2* was used as positive control. MW, marker.

The oncogenic properties of leukemic fusion proteins have already been described in a nude mice model [42]. In order to assess the tumorigenic potential of BCR-JAK2, ten nude mice were subcutaneously injected on the right side above the hind legs with Ba/F3-BCR-JAK2 cells and on the left side with Ba/F3-mock cells. Three mice died of nonrelated events and the rest were analyzed 20 days later, when tumors were clearly observed on the right side in all of them (Figure 6A, left panel). As expected, none of the mice developed tumors on the left side where Ba/F3-mock cells were injected. Considering that cells expressing BCR-JAK2 also co-express EGFP, its expression was confirmed in all tumors using a digital photostation (Figure 6A, right panel). In contrast, no EGFP signal was detected in the flank injected with Ba/F3 mock cells. Tumors were disaggregated and cells were analyzed by flow cytometry, which rendered at least 50% EGFP-expressing cells (Figure 6B). EGFP-negative cells found in those samples could be host accessory tumor cells as connective, epithelial and endothelial cells, although methylation events downregulating EGFP expression on BCR-JAK2-bearing cells could not be excluded [43], [44]. RT-PCR analysis on RNA isolated from three different tumors showed specific amplification of BCR-JAK2 in every case (Figure 6C). We therefore conclude that BCR-JAK2 conferred oncogenic properties to Ba/F3 cells.

### Primary progenitor cells transduced with BCR-JAK2 show increased proliferation

**Figure 7:**
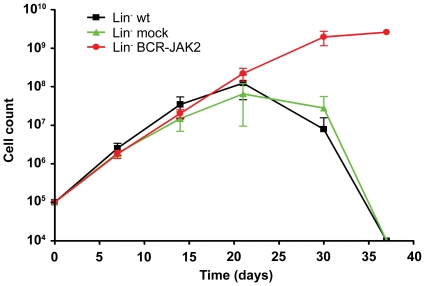
Lin- primary progenitor cells expressing BCR-JAK2 show increased proliferation capacity compared to wild-type (wt) and mock progenitor cells. Lin- progenitor cells obtained from Balb/c mice were transduced with pLZR (mock cells) or pLZR-BCR-JAK2 retroviral particles and seeded for proliferation assay during 30 days.

To test whether mouse primary progenitor cells transduced with BCR-JAK2 have increased survival, transduction of lineage negative (Lin^−^) cells selected from bone marrow of Balb/c mice was performed and in vitro expansion was carried out (Figure 7). Lin^−^ cells transduced with pLZR-BJ showed an increase in proliferation capacity compared to wild type or pLZR-mock transduced cells. In fact, Lin^−^ BCR-JAK2-transduced cells lasted longer than 3 months in vitro (data not shown) while proliferation of control samples decreased after 30 days in culture. These data suggest that besides the proliferative advantage and ability to transform hematopoietic cell lines (Figure 2), BCR-JAK2 increased survival of primary hematopoietic progenitor cells. These findings are in agreement with in vivo transforming abilities as previously described for other leukemogenic proteins [45], [46].

## Discussion

Two-thirds of adult patients with ALL do not have molecular markers associated with the disease; therefore, the search for new markers is an active area of research. We present the unique case of a patient suffering from ALL with a complex karyotype at diagnosis and harboring a *BCR-JAK2* fusion gene. Three additional studies report this translocation in leukemia patients. In contrast with the case presented here, the clinical diagnoses were found in atypical CML [9], [10] and AML [11]. Our finding indicates that this translocation is not restricted to the myeloid lineage. Interestingly, in one of the CML-like cases, the breakpoints in *BCR* and *JAK2* were the same as in our case, whereas in the AML case the breakpoint in *BCR* was similar to that of p210 BCR/ABL and was in exon 11 for *JAK2*. Unlike our case, the patients in the cases mentioned above died after developing a blast crisis.

Specially designed qPCR to detect *BCR-JAK2* is a new tool for molecular diagnosis and follow-up of MRD. In the case presented here, persistence of undetectable MRD correlated with complete genetic and clinical response maintained to the present day. This could be due to the early molecular response achieved prior to SCT [47]. Further studies and more case report data are necessary to determine whether *BCR-JAK2*-positive myeloid/lymphoid neoplasms represent a distinct clinical-pathological entity.

The BCR-JAK2 protein contains the BCR coiled-coil domain fused to the JH1-tyrosine-kinase domain of JAK2. Native JAK2 in normal cells is phosphorylated by oligomerization of growth factor or cytokine receptors, whereas ligand-independent oligomerization of receptor and non-receptor TK in leukemias and solid tumors have been reported [15]. Therefore, in BCR-JAK2, it could be hypothesized that the kinase domain of JAK2 was activated through oligomerization mediated by the coiled-coil domain of BCR, as occurred in the constitutive activation of BCR-ABL [48].

Here, we report that Ba/F3 cells transformed to growth factor-independence by BCR-JAK2 showed STAT5 phosphorylation and translocation into the nucleus. These findings are consistent with the reported activation of the JAK-STAT pathway in transformed BCR-ABL and TEL-JAK2 cells, suggesting an essential role for STAT5 in both cellular transformation and maintenance of disease by TK fusion proteins or the Abelson virus [49], [50], [51]. In normal hematopoietic cells, STAT5 is activated by a wide variety of cytokines that promote differentiation, proliferation, and suppression of apoptosis by regulating expression or phosphorylation of *BcL-2* gene family members [32]. The anti-apoptotic effect mediated by STAT5 activation in hematopoietic progenitors is partially based on the transcriptional regulation of *Bcl-xL* gene expression in response to IL-3 [33] and to BCR-ABL in transformed cells [35], [36]. Consistent with this, we found *Bcl-xL* expression on Ba/F3-BCR-JAK2 cells in the absence of growth factor, suggesting that the JAK2/STAT5/Bcl-xL pathway could be responsible for Ba/F3-BCR-JAK2 cell proliferation and survival. However, we cannot exclude the participation of the BCR moiety in these activation processes [52], [53]. Further studies to evaluate whether different partners could modify the chimeric protein behaviour through specific downstream signal transduction effectors would thus be of interest. It is worth noting that *Bcl-xL* expression was enhanced in the patient samples at diagnosis compared with those taken during remission, as previously described in a variety of human cancers, including AML [54]. Moreover, luciferase assays with HEK293T cells showed that BCR-JAK2 has the ability to induce the Bcl-xL promoter.

Fusion TK's involved in leukemic processes are localized to the cytoplasm, where they maintain activated downstream effectors. Accordingly, we detected BCR-JAK2 protein in the cytoplasm, while pSTAT5 was found in the nucleus of Ba/F3-BCR-JAK2 cells as previously described for Ba/F3 expressing TEL-JAK2 [28].

TG101209-treated BCR-JAK2-expressing cells impaired both endogenous JAK2 and BCR-JAK2 phosphorylation and, consequently, STAT5 activation and *Bcl-xL* expression, all resulting in triggering of apoptosis. Treatment of Ba/F3-mock cells in the presence of IL-3 showed a lower inhibition of wild type JAK2 and STAT5 phosphorylation, as well as Bcl-xL protein expression. Also, apoptosis was lower than in Ba/F3-BCR-JAK2-treated cells, probably due to the anti-apoptotic role reported for IL-3 activation [36].

Finally, the usefulness of immunodeficient mice models to assess the propagation of oncoprotein-expressing cell lines [55], and to confirm their ability to produce tumors has been well established [42]. Here, we have shown how Ba/F3-BCR-JAK2 cells are able to induce tumors in nude mice, thus indicating that BCR-JAK2 has oncogenic properties. Further research will be developed to evaluate whether mouse Lin- progenitors transduced with BCR-JAK2 are able to induce a hematopoietic disease in lethally irradiated recipient mice. Interestingly, this model will also serve as a useful approach for testing new JAK2 inhibitors such as TG101209.

Our results demonstrate that BCR-JAK2 is a hyperactive TK with transforming and tumorigenic properties based on sustained STAT5 activation and *Bcl-xL* induction leading to increased survival. Thus, we hypothesize that BCR-JAK2 could play a central role in the induction of lymphoproliferative disease in this patient. Further studies are necessary to elucidate the incidence of BCR-JAK2 fusion in the development of leukemia in the general population. JAK2 inhibitors abrogate BCR-JAK2 function by blocking the JAK2/STAT5 activation pathway, which in turn leads to cell death by apoptosis. We believe that abnormal fusion transcripts involving *JAK2* should be investigated in patients with atypical CML and acute types of leukemia. JAK2 inhibitors warrant further investigation for use alone or in combination with standard chemotherapy in treating human cancers with elevated JAK2 activity.

## Supporting Information

Figure S1 BCR-JAK2 breakpoint region sequence. (**a**) PCR product sequence (281 bp) obtained at diagnosis with the BCR-ABL primers used for p190 detection. Green, BCR-B primer sequence; black, BCR (exon 1); red, JAK2 (exon 19); blue, complementary sequence of ABL3 primer used for the first PCR amplification. The open reading frame is maintained. (**b**) Detailed sequence alignment analysis of reverse ABL3 primer (blue) showing 14 nucleotides (red) complementary to the flanking region of JAK2 sequence shown in (**a**).
(TIF)

Figure S2 BCR-JAK2 is located in the cytoplasm. Immunofluorescence analysis of HEK293T cells transiently transfected with pLZR carrying BCR-JAK2 (HEK293T-BJ) or control vector (HEK293T-mock) analyzed by confocal microscopy. Transfected cells were detected by EGFP expression. JAK2 expression was shown in red only in the cytoplasm of HEK293T-BJ cells. The merged image showed nuclei stained with DAPI (blue), EGFP (green), and anti-JAK2 Ab (red) on HEK293T-BJ transfected cells. Scale bar = 5 µm.
(TIF)

Figure S3 Quantification of *Bcl-xL* expression in the samples from the patient at diagnosis and at complete molecular remission (CMR) for BCR-JAK2. Bars represent relative *Bcl-xL* levels normalized using *H3* and results are given as mean ± SD (n = 3).
(TIF)

Figure S4 BCR-JAK2 elicited BcL-xL promoter activation. Luciferase assay of HEK293T cells transiently co-transfected with a plasmid bearing the promoter region of BcL-xL tagged to luciferase (pGL2-pmter Bcl-xL 0.6R) together with plasmids coding for either BCR-JAK2 or a constitutively active STAT5. Luciferase activity induction was calculated as firefly/renilla luciferase activities and normalized using control vector-transfected levels. One representative experiment out of three is shown due to the high variation obtained by transient transfection experiments.
(TIF)

Methods S1 (DOC)
