# Supplementary figures and images for: Transforming and Tumorigenic Activity of JAK2 by Fusion to BCR: Molecular Mechanisms of Action of a Novel BCR-JAK2 Tyrosine-Kinase
Source: PLoS One. 2012 Feb 27;7(2):e32451. doi: 10.1371/journal.pone.0032451 (PMC3288102; doi:10.1371/journal.pone.0032451)

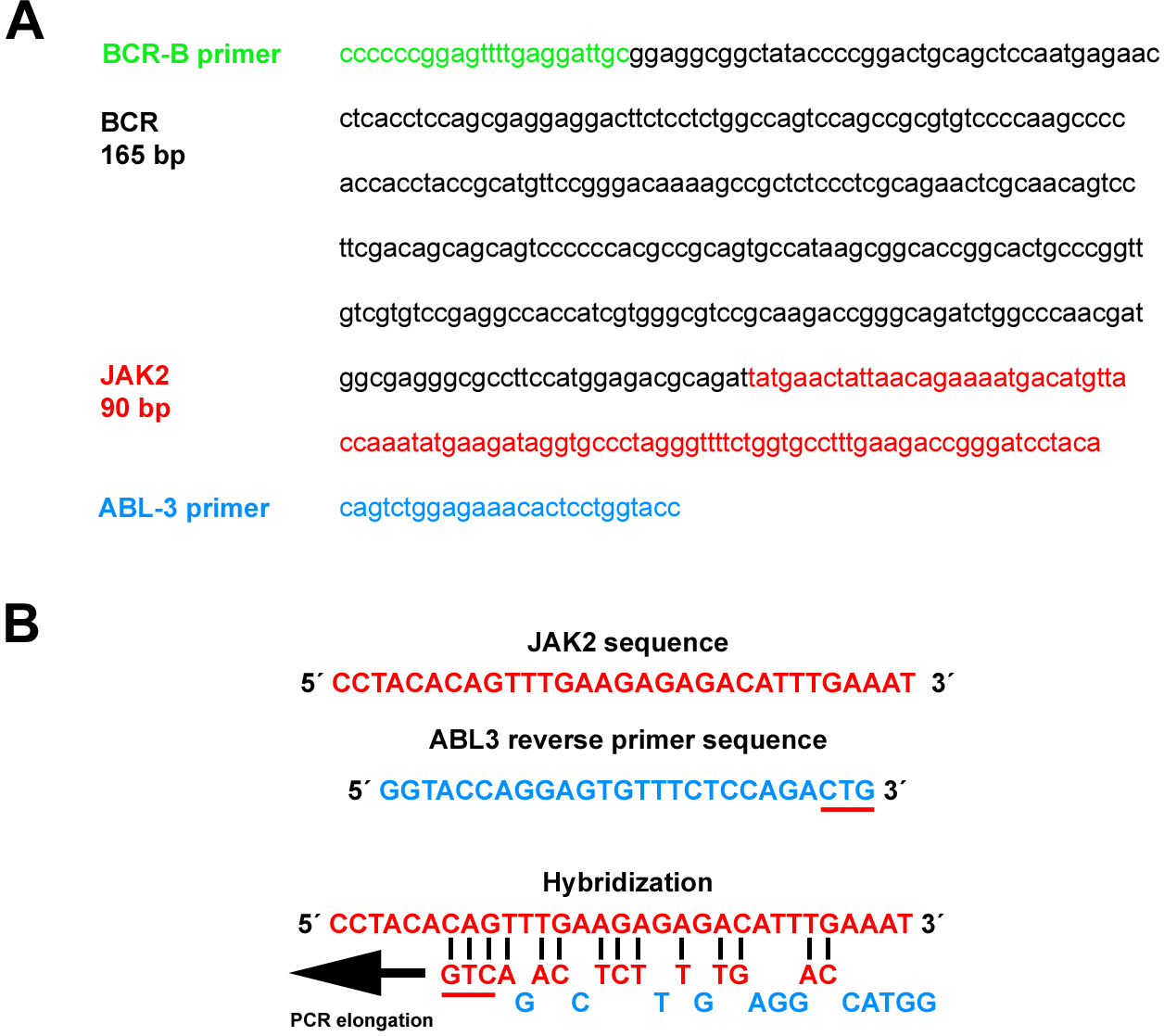

Supplement: Figure S1 — BCR-JAK2 breakpoint region sequence. (a) PCR product sequence (281 bp) obtained at diagnosis with the BCR-ABL primers used for p190 detection. Green, BCR-B primer sequence; black, BCR (exon 1); red, JAK2 (exon 19); blue, complementary sequence of ABL3 primer used for the first PCR amplification. The open reading frame is maintained. (b) Detailed sequence alignment analysis of reverse ABL3 primer (blue) showing 14 nucleotides (red) complementary to the flanking region of JAK2 sequence shown in (a). (TIF) [file pone.0032451.s001.tif]

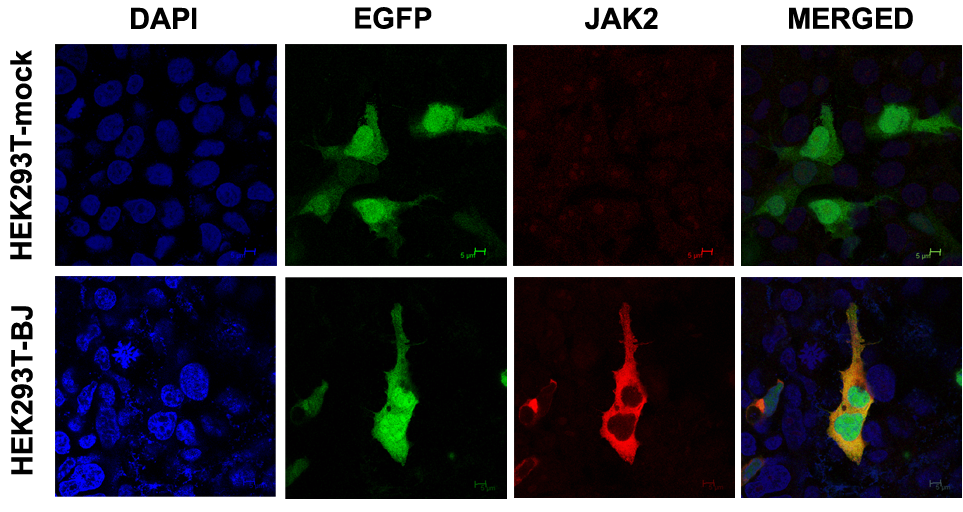

Supplement: Figure S2 — BCR-JAK2 is located in the cytoplasm. Immunofluorescence analysis of HEK293T cells transiently transfected with pLZR carrying BCR-JAK2 (HEK293T-BJ) or control vector (HEK293T-mock) analyzed by confocal microscopy. Transfected cells were detected by EGFP expression. JAK2 expression was shown in red only in the cytoplasm of HEK293T-BJ cells. The merged image showed nuclei stained with DAPI (blue), EGFP (green), and anti-JAK2 Ab (red) on HEK293T-BJ transfected cells. Scale bar = 5 µm. (TIF) [file pone.0032451.s002.tif]

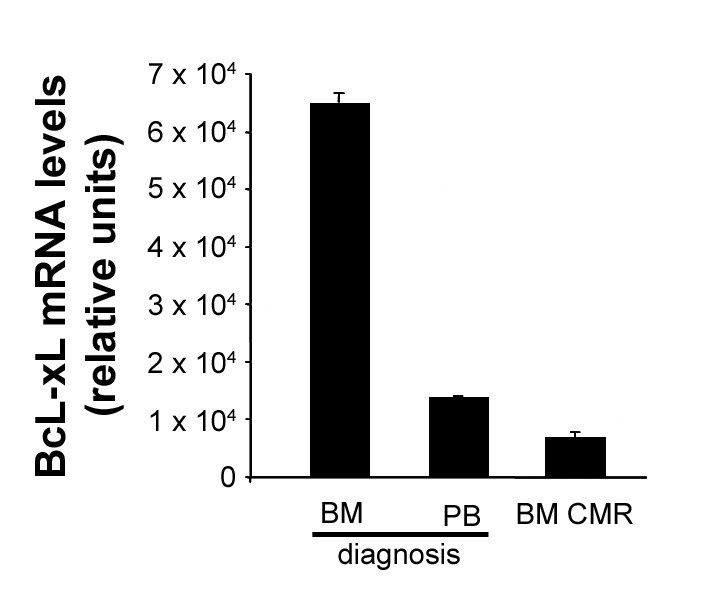

Supplement: Figure S3 — Quantification of Bcl-xL expression in the samples from the patient at diagnosis and at complete molecular remission (CMR) for BCR-JAK2. Bars represent relative Bcl-xL levels normalized using H3 and results are given as mean ± SD (n = 3). (TIF) [file pone.0032451.s003.tif]

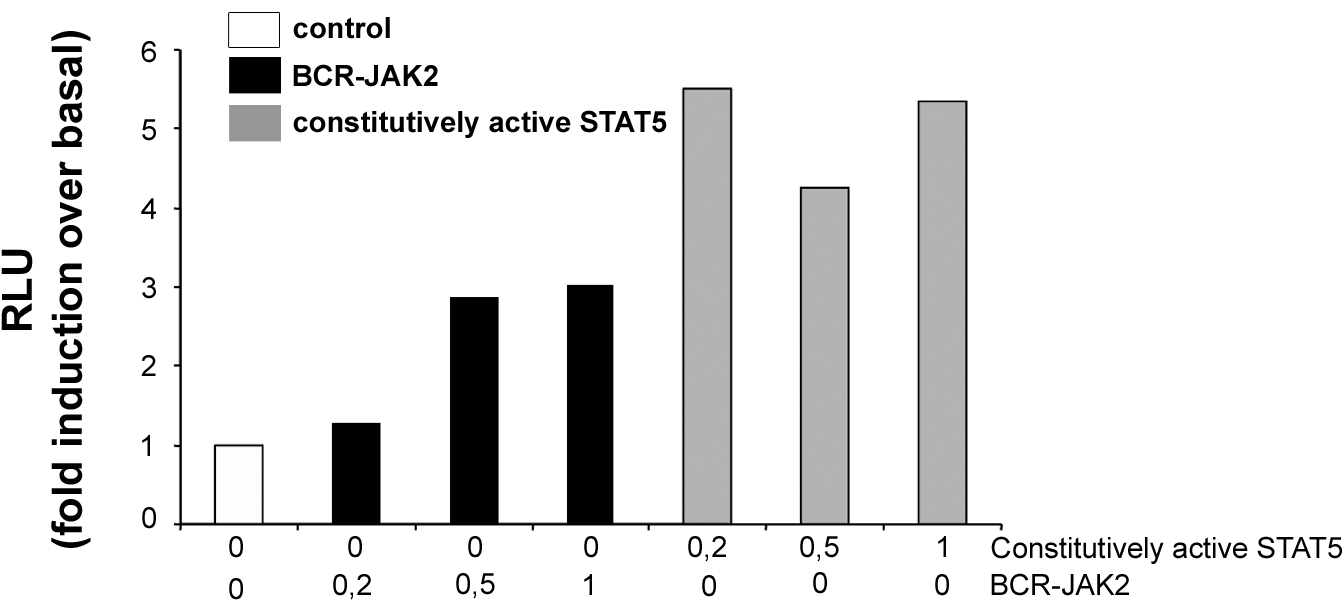

Supplement: Figure S4 — BCR-JAK2 elicited BcL-xL promoter activation. Luciferase assay of HEK293T cells transiently co-transfected with a plasmid bearing the promoter region of BcL-xL tagged to luciferase (pGL2-pmter Bcl-xL 0.6R) together with plasmids coding for either BCR-JAK2 or a constitutively active STAT5. Luciferase activity induction was calculated as firefly/renilla luciferase activities and normalized using control vector-transfected levels. One representative experiment out of three is shown due to the high variation obtained by transient transfection experiments. (TIF) [file pone.0032451.s004.tif]
