# Supplementary material for: Transforming and Tumorigenic Activity of JAK2 by Fusion to BCR: Molecular Mechanisms of Action of a Novel BCR-JAK2 Tyrosine-Kinase
Source: PLoS One. 2012 Feb 27;7(2):e32451. doi: 10.1371/journal.pone.0032451 (PMC3288102; doi:10.1371/journal.pone.0032451)
Supplement: Methods S1 — (DOC) [file pone.0032451.s005.doc]

**SUPPORTING MATERIAL AND METHODS**

**Immunofluorescence**

Transiently transfected HEK293T cells were fixed with 4% paraformaldehyde in PBS for 10 min at room temperature (RT) and permeabilized with 0.1% NP-40 in PBS. Cells were blocked with TNB (0.1 M Tris-HCl, 0.15 M NaCl and 0.5% blocking reagent [Boehringer Mannheim]) for 45 min at 37ºC and incubated with anti-JAK2 antibody (C-20 Ab) (Santa Cruz Biotechnology, CA, USA) for 2 hours at 37ºC. After washing, cells were incubated with a secondary goat anti-mouse Rhodamine Red™-X (H+L) Ab (Molecular Probes) for 25 min at 37ºC. Nuclei were stained with DAPI (4´, 6-diamidine-2´-phenylindole, dihydrochloride, Pierce). The cover slips were mounted with DakoCytomation Mounting Medium (Dako). Preparations were analyzed using a Leica TCS-Sp (Leica Microsystems) confocal microscope.

**Luciferase reporter assay**

HEK293T cells were transfected with a mixture of 2 µg of a luciferase reporter vector bearing a 0.6-kb fragment of the mouse *Bcl-xL* promoter containing a STAT-binding element (pGL2-pmter Bcl-xL 0.6R)^1,2^ (200 ng/10^5^ cells) and pRL-Null (15 ng/10^5^ cells) (Promega) bearing a promoterless *Renilla* luciferase gene used to normalize all the firefly-luciferase values obtained. The reporter plasmids were transfected together with 0.2, 0.5, or 1 µg of pCDNA3.1-BCR-JAK2 expression vector or empty pCDNA3.1 vector (Invitrogen) with Fugene HD (Roche) according to the manufacturer’s instructions. In some cases, the pCDNA-STAT5▲750STAT6JAK2 construct was used as a constitutively active STAT5.^3^ Luciferase activity was determined 48 h after transfection with the Dual-Luciferase Reporter Assay System (Promega) in a luminometer (Berthold Detection Systems) and expressed as relative light units (RLU). All experiments were carried out in duplicate and data are presented as relative values.

**REFERENCES**

1. Boise LH, González-García M, Postema CE, Ding L, Lindsten T, et al. (1993) Bcl-x, a bcl-2-related gene that functions as a dominant regulator of apoptotic cell death. Cell 74: 597-608.

2. Silva M, Benito A, Sanz C, Prosper F, Ekhterae D, et al. (1999) Erythropoietin can induce the expression of bcl-x(L) through Stat5 in erythropoietin-dependent progenitor cell lines. J Biol Chem 274: 22165-22169.

3. Berchtold S, Moriggl R, Gouilleux F, Silvennoinen O, Beisenherz C, et al. (1997) Cytokine receptor-independent, constitutively active variants of STAT5. J Biol Chem 272: 30237-30243.
